# Excited-state spin-resonance spectroscopy of V${}_{{{{{{{{\rm{B}}}}}}}}}^{-}$ defect centers in hexagonal boron nitride

**DOI:** 10.1038/s41467-022-30772-z

**Published:** 2022-06-09

**Authors:** Nikhil Mathur, Arunabh Mukherjee, Xingyu Gao, Jialun Luo, Brendan A. McCullian, Tongcang Li, A. Nick Vamivakas, Gregory D. Fuchs

**Affiliations:** 1 School of Applied and Engineering Physics, Cornell University, Ithaca, NY USA; 2 The Institute of Optics, University of Rochester, Rochester, NY USA; 3 Department of Physics and Astronomy, Purdue University, West Lafayette, IN USA; 4 Department of Physics, Cornell University, Ithaca, NY USA; 5 Elmore Family School of Electrical and Computer Engineering, Purdue University, West Lafayette, IN USA; 6 Materials Science, University of Rochester, Rochester, NY USA; 7 Department of Physics and Astronomy, University of Rochester, Rochester, NY USA; 8 Center for Coherence and Quantum Optics, University of Rochester, Rochester, NY USA; 9 Kavli Institute at Cornell for Nanoscale Science, Ithaca, NY USA

**Keywords:** Qubits, Two-dimensional materials, Single photons and quantum effects

## Abstract

The recently discovered spin-active boron vacancy (V${}_{{{{{{{{\rm{B}}}}}}}}}^{-}$) defect center in hexagonal boron nitride (hBN) has high contrast optically-detected magnetic resonance (ODMR) at room-temperature, with a spin-triplet ground-state that shows promise as a quantum sensor. Here we report temperature-dependent ODMR spectroscopy to probe spin within the orbital excited-state. Our experiments determine the excited-state spin Hamiltonian, including a room-temperature zero-field splitting of 2.1 GHz and a g-factor similar to that of the ground-state. We confirm that the resonance is associated with spin rotation in the excited-state using pulsed ODMR measurements, and we observe Zeeman-mediated level anti-crossings in both the orbital ground- and excited-state. Our observation of a single set of excited-state spin-triplet resonance from 10 to 300 K is suggestive of symmetry-lowering of the defect system from *D*_3*h*_ to *C*_2*v*_. Additionally, the excited-state ODMR has strong temperature dependence of both contrast and transverse anisotropy splitting, enabling promising avenues for quantum sensing.

## Abstract (web summary)

The negatively charged boron vacancy in hBN shows promise as a quantum sensor, but, until recently, the focus has been on its ground-state properties. Here, the authors report temperature-dependent spin-resonance optical spectroscopy of the orbital excited state.

## Introduction

Optically addressable, spin-active defects and quantum dots in the solid-state have emerged as promising qubits and quantum sensors^1–3^ because robust control techniques enable facile quantum gates and sensing protocols^4^. The recent advent of two-dimensional (2D) materials has stimulated the search for spin-active defects that can be integrated into van der Waals heterostructures, enabling a wide array of optoelectronic and nano-photonic devices that take advantage of their optical and spin properties^5–14^. A spin-active defect in a 2D material is especially promising for nanoscale sensing of interfacial phenomena with high sensitivity due to narrow spin transition linewidths and the ability to position these atomic-scale systems at sub-nanometer distances from the surface of a sample^15,16^.

Interestingly, the wide bandgap 2D material hexagonal boron nitride (hBN), which has been known to host bright and stable single-photon emitting defects^17–22^, is now also known to host spin-active defects that are addressable at room temperature^23–27^. Significant progress has been made in understanding the spin-active orbital ground-state (GS) of the negatively charged boron vacancy (V${}_{{{{{{{{\rm{B}}}}}}}}}^{-}$) defect, which is an orbital-singlet and spin-triplet with zero-field electron spin-resonance at 3.5 GHz arising from spin–spin interactions^23^. However, there are only tentative proposals for the energy level structure of the excited-state (ES) as well as the overall symmetry of the defect, without experimental confirmation^28–30^. The highest point group symmetry of the V${}_{{{{{{{{\rm{B}}}}}}}}}^{-}$ defect is D_3*h*_, with allowed optical transitions between the ${}^{3}{A}_{2}^{\prime}$ GS and the ^3^*E″* ES^29,30^, but it is expected that symmetry breaking due to strain may result in a lowered *C*_2*v*_ symmetry that will lift the two-fold orbital degeneracy initially present in the D_3*h*_ system. In comparison to the nitrogen-vacancy (NV^−^) center in diamond, where careful understanding of the ES Hamiltonian^31–36^ was instrumental for key advances including spin readout enhancement^37^, nuclear spin polarization^38,39^, opto-mechanical spin control^40^, and spin-photon entanglement^3^, it is expected that understanding the ES of V${}_{{{{{{{{\rm{B}}}}}}}}}^{-}$ in hBN will be critical to unlocking its potential for quantum technologies.

In this work, we perform temperature-dependent continuous-wave (CW) and pulsed optically detected magnetic resonance (ODMR) measurements to manipulate the electronic spin of V${}_{{{{{{{{\rm{B}}}}}}}}}^{-}$ defects in hBN and reveal the orbital excited-state Hamiltonian. Using confocal microscopy, we excite defect ensembles in multilayer flakes of hBN with a 532 nm laser, and collect the emitted photoluminescence (PL) around ${\lambda }_{\max }\approx 800$ nm (Supplementary Fig. 1) as a function of applied microwave (MW) frequency to determine the electron spin resonance (ESR) spectrum. At room temperature, we measure a zero-field longitudinal splitting *D*_es_ of 2.1 GHz, transverse splitting *E*_es_ of 74 MHz, and a Landé g-factor of 2. Our findings explain the magnetic-field-dependent photoluminescence in terms of Zeeman-mediated level anti-crossings in both the ground- and excited-state spin manifolds. In addition, our temperature-dependent ODMR spectra show that, unlike the NV^−^ center in diamond^32^, the spin-resonance contrast in the ES persists at low temperatures, suggesting that the ES is an orbital singlet. We observe linewidth narrowing and contrast enhancement as the temperature is lowered, which is consistent with previous reports of the temperature-dependent ES lifetime^41^.

## Results

**Fig. 1:**
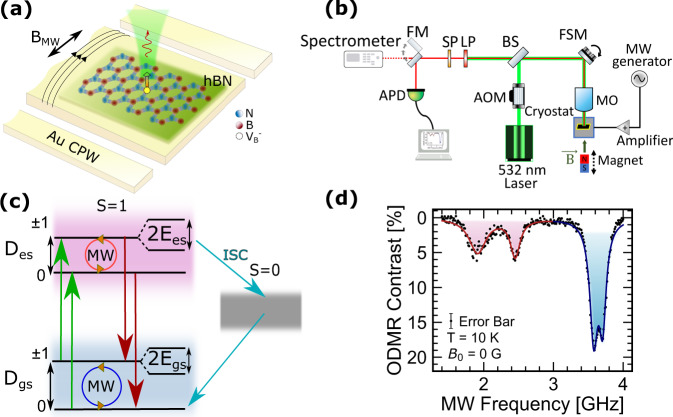
Spin-active V${}_{{{{{{{{\rm{B}}}}}}}}}^{-}$ defects in hBN. **a** Schematic of the device used to probe spin transitions of V${}_{{{{{{{{\rm{B}}}}}}}}}^{-}$ defects in hBN. hBN flakes are transferred onto a gold-film coplanar waveguide (CPW) and the generated microwave field *B*_MW_ induces rotations of the defect spin state which is read out via PL. **b** Schematic of the experimental setup. AOM acousto-optic modulator, BS beam splitter, FSM fast-steering mirror, MO microscope objective, LP long-pass filter, SP short-pass filter, FM flip mirror, APD avalanche photodiode. **c** Energy level diagram of the defect orbital states and their spin sublevels, which are split by *D*_es_ and *D*_gs_ in the ES and GS, respectively. A non-radiative ISC to a singlet state is preferred from the $\left|\pm 1\right\rangle$ spin sublevels of the ES. **d** Zero-field ODMR spectrum at *T* = 10 K, excited with laser power *P*_L_ ≈ 3 mW and microwave power *P*_MW_ ≈ 160 mW, showing distinct resonance dips from spin transitions in the ES (red) and GS (blue) at their respective splitting frequencies. The error bar represents shot noise from photon counting.

The V${}_{{{{{{{{\rm{B}}}}}}}}}^{-}$ defect in hBN has a spin-triplet in both the orbital GS and ES^23,30^, with the electronic spin oriented out-of-plane with respect to the hexagonal crystal lattice of the hBN. Spin–spin interactions introduce a zero-field splitting between the $\left|{m}_{{{{{{{{\rm{S}}}}}}}}}=0\right\rangle$ and $\left|{m}_{{{{{{{{\rm{S}}}}}}}}}=\pm 1\right\rangle$ spin states, where *m*_S_ is the spin quantum number. The room temperature zero-field longitudinal splitting of the GS, *D*_gs_, has been measured to be ~3.5 GHz^23,41^, but the splitting in the excited state, *D*_es_, was previously unknown. Optical transitions between orbital states are spin-conserving, with the exception of a non-radiative relaxation mechanism through an inter-system crossing (ISC) to a spin-singlet state that is selectively preferred from $\left|{m}_{{{{{{{{\rm{S}}}}}}}}}=\pm 1\right\rangle$ in the ES (Fig. 1c). This non-radiative relaxation process results in a measurable difference in photoluminescence (PL) intensity, allowing for optical readout of the defect spin state.

To probe the spin transitions of the V${}_{{{{{{{{\rm{B}}}}}}}}}^{-}$ center, we fabricate devices illustrated by the schematic in Fig. 1a. First, multilayer hBN flakes are mechanically exfoliated onto a silicon substrate with a thermal oxide layer and He^+^ ion implantation is used to break B–N bonds, creating spin-active V${}_{{{{{{{{\rm{B}}}}}}}}}^{-}$ defects. We use photolithography to pattern a gold-film coplanar microwave waveguide on a sapphire substrate. Then, hBN flakes with spin-active defects are lifted from the SiO_2_/Si substrate with a polycarbonate (PC) stamp and transferred directly onto the central conductor of the waveguide so that the generated microwave magnetic field, ${\overrightarrow{B}}_{{{{{{{{\rm{MW}}}}}}}}}$, is orthogonal to the defect spin axis. ODMR is performed by sweeping the frequency of *B*_MW_ while optically pumping with a 532 nm laser and measuring the photoluminescence intensity (*I*_PL_) with an avalanche photodiode (APD) (Fig. 1b). When *B*_MW_ is not resonant with spin transitions, the defect remains spin-polarized in the $\left|{m}_{{{{{{{{\rm{S}}}}}}}}}=0\right\rangle$ spin state and emits the maximum value of *I*_PL_. However, on resonance, the spin is rotated toward $\left|{m}_{{{{{{{{\rm{S}}}}}}}}}=\pm 1\right\rangle$ and *I*_PL_ is reduced due to the enhancement in non-radiative relaxation through the ISC. From the initial state of $\left|{m}_{{{{{{{{\rm{S}}}}}}}}}=0\right\rangle$ in the orbital GS, the defect can relax through the ISC if either the spin has been rotated toward the $\left|{m}_{{{{{{{{\rm{S}}}}}}}}}=\pm 1\right\rangle$ spin sublevels in the GS and then optically excited, or if it is first optically excited and then rotated while in the ES. Therefore, under continuous MW driving and optical pumping, spin transitions can be induced in both the GS and ES, which is evident in the low-temperature zero-field ODMR spectrum shown in Fig. 1d. Lorentzian fits to the data are shown in separate colors to distinguish the GS (blue) and ES (red) resonances centered at *D*_gs_ ± *E*_gs_ and *D*_es_ ± *E*_es_.

Due to the Zeeman effect, the degeneracy of the $\left|{m}_{{{{{{{{\rm{S}}}}}}}}}=\pm 1\right\rangle$ states is lifted by an external magnetic field $\overrightarrow{{B}_{0}}$. Similarly to the ground-state^23^, the excited-state spin structure can be described by the Hamiltonian

1 $${\hat{H}}_{{{{{{{{\rm{es}}}}}}}}}={\hat{H}}_{0}+\underbrace{h{D}_{{{{{{{{\rm{es}}}}}}}}}\left({\hat{{S}_{z}}}^{2}-\frac{S(S+1)}{3}\right)}_{\begin{array}{c}{{{{{{{\rm{longitudinal}}}}}}}}\,{{{{{{{\rm{splitting}}}}}}}}\end{array}}+\underbrace{h{E}_{{{{{{{{\rm{es}}}}}}}}}\left({\hat{{S}_{x}}}^{2}-{\hat{{S}_{y}}}^{2}\right)}_{\begin{array}{c}{{{{{{{\rm{transverse}}}}}}}}\,{{{{{{{\rm{splitting}}}}}}}}\end{array}}+\underbrace{{\mu }_{B}{g}_{{{{{{{{\rm{es}}}}}}}}}{\overrightarrow{B}}_{0}\cdot \hat{\overrightarrow{S}}}_{\begin{array}{c}{{{{{{{\rm{Zeeman}}}}}}}}\,{{{{{{{\rm{interaction}}}}}}}}\end{array}}+\underbrace{{\hat{H}_{{{{{{{{\rm{HF}}}}}}}}}}}_{\begin{array}{c}{{{{{{{\rm{hyperfine}}}}}}}}\end{array}}$$

where ${\hat{H}}_{0}$ is the dominant electronic term giving the energy with respect to the GS, *h* is Planck’s constant, *μ*_*B*_ is the Bohr magneton, *g*_es_ is the ES Landé g-factor, {${\hat{S}}_{x},{\hat{S}}_{y},{\hat{S}}_{z}$} are the components of the spin operator $\hat{\overrightarrow{S}}$, and S = 1 for spin-triplet levels. To study ODMR of Zeeman-split states, we use a permanent magnet aligned perpendicular to the waveguide plane to introduce a static magnetic field. In the limit of a perfectly flat and conformal hBN layer, the field direction will closely coincide with the c-axis of the hBN crystal. From Eq. (1), the field magnitude *B*_0_ shifts the ES spin transition frequencies of the $\left|{m}_{{{{{{{{\rm{S}}}}}}}}}=\pm 1\right\rangle$ states to

2 $${\nu }_{\pm }={D}_{es}\pm \sqrt{{{E}_{es}}^{2}+{\left({\mu }_{B}{g}_{{{{{{{{\rm{es}}}}}}}}}{B}_{0}/h\right)}^{2}}$$

**Fig. 2:**
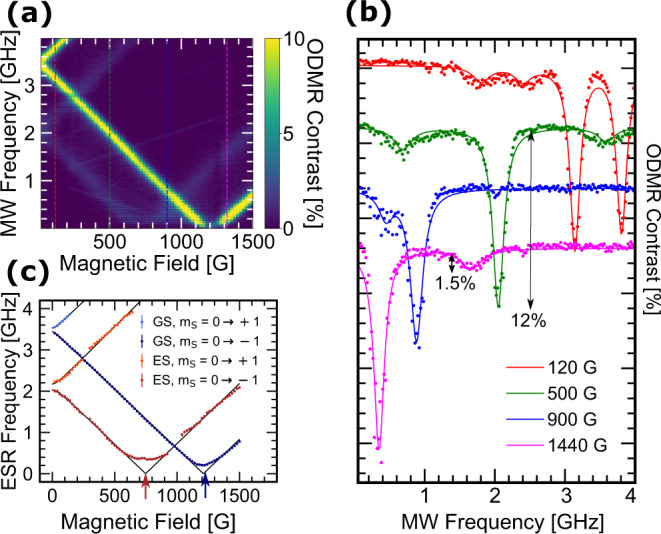
Room temperature field dependence of CW ODMR and spin resonance frequencies. **a** Magnetic-field dependence of the CW ODMR contrast at room temperature. **b** Individual ODMR traces and their fits at four values of *B*_0_ indicated by the vertical colored dashed lines in **a**. **c** Field dependence of the fitted ODMR peaks corresponding to the electron spin resonance (ESR) frequencies. Error bars represent the standard deviation of the fitted parameter. Fits to Eq. (2) are shown in black. The field magnitudes at which the $\left|0\right\rangle$ and $\left|-1\right\rangle$ spin states cross in energy are marked with red and blue arrows.

Figure 2a shows the room-temperature magnetic-field-dependent ODMR spectra of the defect ensemble from 0 to 1500 G, at microwave power *P*_MW_ ≈ 100 mW. Line cuts at four values of *B*_0_ are shown in Fig. 2b along with their fitted curves. The known resonant dips from the $\left|{m}_{{{{{{{{\rm{S}}}}}}}}}=0\right\rangle \to \left|{m}_{{{{{{{{\rm{S}}}}}}}}}=\pm 1\right\rangle$ transitions in the GS^23,26^ are present, as well as the additional unreported dips that shift with the applied field along paths parallel to the GS resonance lines, which we attribute to spin transitions in the ES.

To quantify the electron spin resonance (ESR) transition frequencies, we fit each ODMR trace with the appropriate number of Lorentzians, and plot the resonant frequencies as a function of applied field (Fig. 2c). Note that because the ODMR contrast of the ES resonances are significantly lower than those of the GS, we are not able to accurately fit the ES ESR frequencies around values of *B*_0_ where the two overlap, and thus there are some gaps in the ES data. We fit the ESR data with Eq. (2) to extract the Hamiltonian parameters. Our measurements agree with previously reported^16,23,26,41^ GS splitting parameters *D*_gs_ = 3.48 ± 0.02 GHz and *E*_gs_ = 48.0 ± 7.1 MHz, and establish the ES splitting parameters *D*_es_ = 2.11 ± 0.03 GHz and *E*_es_ = 74 ± 42 MHz. We also observe that *g*_es_ ≈ *g*_gs_ ≈ 2, which indicates that the orbital angular momentum does not play a significant role in the ES spin structure. The average ES linewidth over the field sweep at this microwave power is 133 ± 32 MHz. These measurements were taken at multiple locations from three different hBN flakes with repeatable results. We do not resolve hyperfine splitting in the ES possibly due to power broadening, the short ES lifetime, and inhomogenous broadening in the defect ensemble. Based on the observed linewidth, we estimate an upper limit of ~100 MHz for the ES hyperfine splitting.

**Fig. 3:**
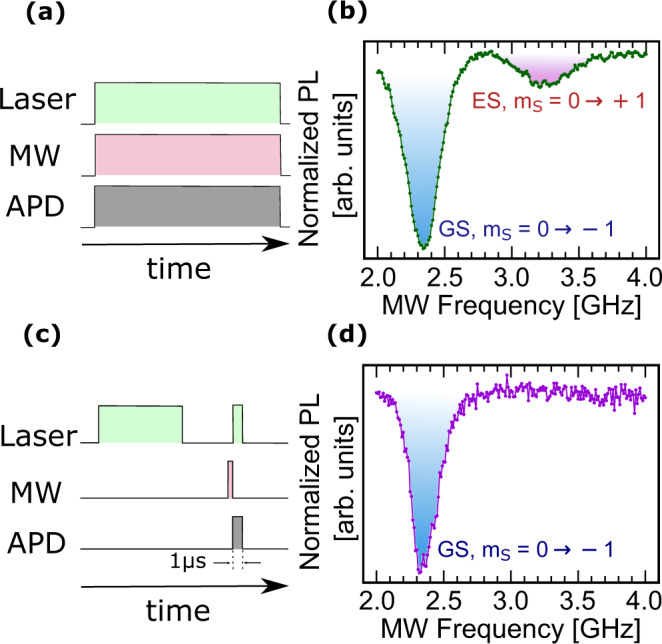
Pulsed ODMR at ***B***_**0**_ = 400 G. **a** CW ODMR is performed with the pumping laser, *B*_MW_, and avalanche photodiode (APD) photon counters on at all times. **b** The resulting CW ODMR spectrum shows spin transitions in both the GS (blue) and ES (red). **c** Pulsed ODMR is performed with *B*_MW_ on only when the laser is off, after the defect is initialized to $\left|{m}_{{{{{{{{\rm{S}}}}}}}}}=0\right\rangle$ in the GS. As soon as *B*_MW_ is turned off, the laser and photon counters are then turned on to read out the spin state. **d** The resulting pulsed ODMR spectrum indicates spin rotation only in the GS, confirming that the additional broader resonance is from an ES transition.

To verify our attribution of the additional ESR frequencies to spin transitions within the orbital excited-state, we implement a pulsed ODMR sequence to compare with CW ODMR results. In the CW measurement, we apply simultaneous microwave and optical excitation (Fig. 3a), allowing spin rotations to occur both in the GS and in the short time interval spent in the ES, resulting in ODMR contrast at the GS and ES ESR frequencies. In the pulsed measurement, the signals are timed such that the microwave pulse trails the optical excitation pulse by a delay that is much longer than the ES lifetime (1–2 ns) (Fig. 3c). In this case, the ES population is negligible when the microwave fields are applied and consequently, the ODMR contrast at the ES ESR frequency disappears if one of the observed spin transitions occurs in the ES. We achieve this by pulsing the laser excitation for 20 μs followed by a waiting period of 5 μs to ensure that the V${}_{{{{{{{{\rm{B}}}}}}}}}^{-}$ defects have relaxed to the orbital GS with a $\left|{m}_{{{{{{{{\rm{S}}}}}}}}}=0\right\rangle$ spin polarization. While in the dark, we apply a 500 ns burst of *B*_MW_, followed immediately by a 1 μs laser pulse and photon collection for readout. We sweep the frequency of *B*_MW_ from 2 to 4 GHz with a step size of 10 MHz and repeat the measurement ~10^4^ times at each frequency to build statistics. The results of the CW and the pulsed ODMR measurements at *B*_0_ = 400 G are shown in Fig. 3b and d, respectively. In the CW ODMR spectrum, we observe a $\left|{m}_{{{{{{{{\rm{S}}}}}}}}}=0\right\rangle \to \left|{m}_{{{{{{{{\rm{S}}}}}}}}}=-1\right\rangle$ resonance in the GS at ~2.3 GHz and a $\left|{m}_{{{{{{{{\rm{S}}}}}}}}}=0\right\rangle \to \left|{m}_{{{{{{{{\rm{S}}}}}}}}}=+1\right\rangle$ resonance in the ES at ~3.3 GHz. In the pulsed ODMR spectrum, we only see the GS resonance at ~2.3 GHz, confirming that the resonance at ~3.3 GHz indeed belongs to the excited-state manifold^31^.

Our observations demonstrate that only a single set of spin-triplet ES resonances (Fig. 3a) exists within the measured frequency range. The lack of a second set of spin transitions in the ES has consequences for understanding the symmetry of this defect. Electronic orbitals are determined by the irreducible representations of the point group symmetry of the defect, and theoretical predictions of the V${}_{{{{{{{{\rm{B}}}}}}}}}^{-}$ center suggest that it has optical excited states of either *D*_3*h*_ symmetry with ^3^*E″* orbitals, or *C*_2*v*_ symmetry with ^3^*B*_1_ and ^3^*A*_1_ excited states^28–30^. One possibility is that we have overlapping spin resonances for doubly-degenerate E states which will be indistinguishable in our measurements. Alternatively, it is plausible that the symmetry is indeed lowered to *C*_2*v*_ and the observed set of spin-triplet resonances belongs to one of the two energy-split orbitals. The spin resonances of the other orbitals can potentially lie beyond the measured frequency range. In what follows, we perform temperature-dependent ODMR and magnetic-field-dependent PL measurements to explore each of these possibilities.

In previous investigations of systems like the ^3^*E* ES of the diamond NV^−^ center, the dynamic Jahn–Teller effect averages the two orbital branches at room temperature, but at low temperatures, the effect is quenched and the ODMR contrast at MW frequency *D*_es_ diminishes^32,33^. To look for signatures of this effect in V${}_{{{{{{{{\rm{B}}}}}}}}}^{-}$ centers in hBN, we cool our device in an optical cryostat. Two key observations at lowered temperatures indicate symmetry lowering. First, we observe that the transverse anisotropy splitting drastically increases from 74 MHz at room temperature to 258 ± 2 MHz, an effect that is not observed in the GS^41^. This is possibly due to strain induced by the difference in thermal expansion of the hBN flake and the substrate, which supports the assumption of symmetry lowering^28–30^. Second, we find that the ES ODMR contrast not only persists down to *T* = 10 K, but in fact increases in magnitude which is unlike previous reports on ^3^*E* ES of NV^−^ centers in diamond^32,33^. The absence of ODMR contrast quenching is consistent with lifting of the double-degeneracy of ^3^*E* states leading to split orbitals with a high enough energy gap rendering the system insensitive to dynamic Jahn–Teller distortion effects.

**Fig. 4:**
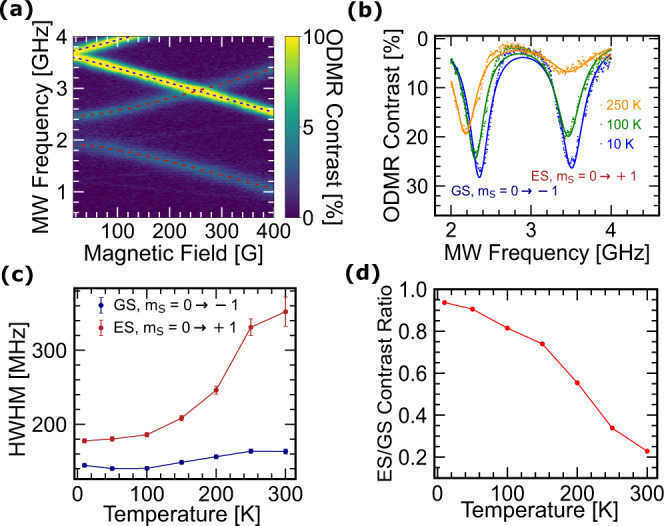
Temperature dependence of the ES ESR. **a** Magnetic-field-dependent CW ODMR at *T* = 10 K and *P*_MW_ ≈ 50 mW. ESR fits to Eq. (2) have been marked with blue and red dashed lines, respectively. **b** CW ODMR spectra at *T* = 250 K (orange), 100 K (green), and 10 K (blue) at *B*_0_ = 450 G and *P*_MW_ ≈ 2.51 W. **c** Temperature dependence of the ESR linewidths for both the GS (blue) and ES (red) at *P*_MW_ ≈ 2.51 W. Error bars represent the standard deviation of the fitted parameter. **d** Relative contrast of the ES ESR with respect to the GS ESR contrast at *P*_MW_ ≈ 2.51 W.

The increased transverse anisotropy splitting is visible in Fig. 4a, where we show the CW ODMR at 10 K as we sweep *B*_0_ from 0 to 400 G at *P*_MW_ ≈ 50 mW. The dashed blue and red curves indicate the fits of the field-dependent ESR frequencies to Eq. (2) for the GS and ES, respectively. Next we apply a magnetic field of *B*_0_ = 450 G and sweep *P*_MW_ at several temperatures from 10 to 300 K (Supplementary Fig. 2). In Fig. 4b, we show the ODMR spectra at three temperatures measured at *P*_MW_ = 2.51 W. From these measurements, it is apparent that the ODMR contrast is more sensitive to temperature in the ES than in the GS. We find that while the GS ESR linewidth is relatively insensitive to temperature, the ES linewidth decreases with temperature (Fig. 4c). This trend in the data is consistent with previous reports of the temperature-dependent ES lifetime^41^. Most notably, we observe a dramatic enhancement of the ES ODMR contrast at low temperature, approaching the same contrast as the GS ODMR at saturated *P*_MW_ (Fig. 4d). This is also consistent with a longer ES lifetime at low temperature because a longer average lifetime allows the ES spin to accumulate more rotation toward $\left|{m}_{{{{{{{{\rm{S}}}}}}}}}=\pm 1\right\rangle$ at a given Rabi frequency (${{\Omega }}\propto \sqrt{{P}_{{{{{{{{\rm{MW}}}}}}}}}}$). Given that the overall excited state lifetime is ~2 ns at 10 K^41^, observation of this effect requires Ω ≈ 100 MHz. To confirm our explanation of the temperature-dependent contrast, we crudely model the on-resonance ODMR using $\langle {{\Delta }}{I}_{{{{{{{{\rm{PL}}}}}}}}}\rangle ={{\Delta }}C{\sin }^{2}(\frac{{{\Omega }}}{2}{\tau }_{{{{{{{{\rm{es}}}}}}}}})$, where 〈Δ*I*_PL_〉 is the normalized PL change measured in the defect ensemble, Δ*C* is the temperature-independent maximum ESR contrast at saturation (~25% at *P*_MW_ ≈ 2.51 W), Ω is the Rabi frequency, and *τ*_es_ is the temperature-dependent ES lifetime. Using reported ES lifetime values^41^, our model predicts that the absolute ES ODMR contrast at *T* = 300 K is ~10%, which agrees well with our observations (Supplementary Notes 3).

Next, we study the influence of magnetic field on the fluorescence of V${}_{{{{{{{{\rm{B}}}}}}}}}^{-}$ defects at room temperature without microwave excitation. At magnetic fields where the $\left|{m}_{{{{{{{{\rm{S}}}}}}}}}=0\right\rangle$ and $\left|{m}_{{{{{{{{\rm{S}}}}}}}}}=-1\right\rangle$ levels cross each other in energy, spin mixing occurs if the defect axis and magnetic field direction are not perfectly aligned (Supplementary Fig. 3). The off-axis component of $\overrightarrow{{B}_{0}}$ causes a level anti-crossing (LAC), making the spin eigenstates a mixture of $\left|{m}_{{{{{{{{\rm{S}}}}}}}}}=0\right\rangle$ and $\left|{m}_{{{{{{{{\rm{S}}}}}}}}}=-1\right\rangle$. Because non-radiative orbital relaxation is more efficient for $\left|{m}_{{{{{{{{\rm{S}}}}}}}}}=-1\right\rangle$ than for $\left|{m}_{{{{{{{{\rm{S}}}}}}}}}=0\right\rangle$, mixed spin states have reduced PL intensity, which has been observed in NV^−^ centers in diamond^42^. Using Eq. (2) and our measured values of *D*_es_ and *D*_gs_, we expect the excited- and ground-state level anti-crossings (denoted by ELAC and GLAC henceforth) to occur at *B*_0_ ≈ 750 G and 1240 G, respectively. These fields are marked with red and blue arrows in Fig. 2c.

**Fig. 5:**
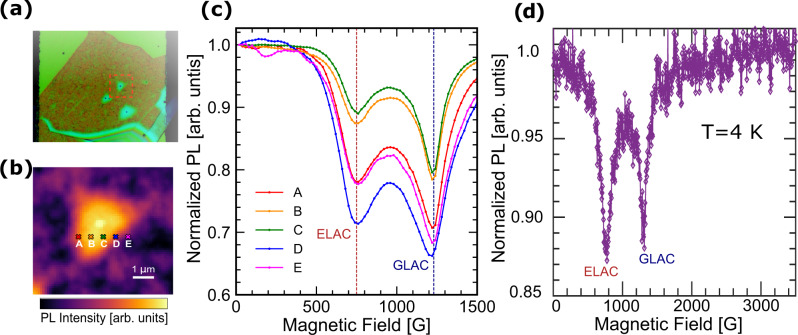
Magnetic-field-dependent PL and level avoided crossings. **a** Optical micrograph of an hBN flake on the Au CPW. Micro-bubbles are formed during the transfer process, from which we can study the effect of the magnetic-field angle on the level anti-crossings (LACs). **b** Spatially resolved PL intensity map of the region indicated by the red dashed box in **a**. **c** Field-dependent PL measurements collected at the corresponding marked locations in **b** show reduction in PL at the ELAC and GLAC field values predicted by our CW ESR results. Variation across locations around the micro-bubble indicate that more spin mixing occurs at greater angles with respect to $\overrightarrow{{B}_{0}}$**. d** Field-dependent PL upto 3500 G. No additional LACs are evident.

In Fig. 5a–c, we show field-dependent PL measurements at room temperature and in the spatial vicinity of a micro-bubble formed during the transfer process of the hBN flake. Even though the field direction is fixed, we expect that defects at different locations on the bubble will have their defect axes oriented in slightly different directions and thus have different angles with respect to $\overrightarrow{{B}_{0}}$. Figure 5a shows an optical micrograph of one of our devices. A spatially resolved PL intensity map of the region indicated by the red dashed box is shown in Fig. 5b. The bright region is a micro-bubble and the locations of further measurements are indicated by colored markers (labeled A–E). The magnetic-field-dependent PL measurements at each of these locations are shown in Fig. 5c and plotted in the corresponding colors. We see two minima that we assign to the ELAC and GLAC. The magnetic field magnitudes for the ELAC and GLAC calculated above agree well with our observations (Fig. 5c). The PL reduction magnitude varies with location around the bubble, as expected due to the non-uniform topography of the hBN flake^42^. To underscore the repeatability of our results, we also performed field-dependent PL extending to even higher fields at *T* = 4 K (Fig. 5d) on another hBN flake. This allows us to investigate the presence of additional LACs arising from spin-resonances with D parameters beyond our probed frequency range. We did not find any evidence of additional LACs upto 3.5 kG of applied field. Assuming a g-factor of g ~ 2, this measurement probes for a LAC in a spin-triplet manifold to a maximum D parameter of ~10 GHz. Thus, our data suggest the absence of additional ES and GS spin manifolds having D < 10 GHz that participate in triplet-singlet inter-system crossings. These observations further corroborate our understanding of the ES and GS spin-dependent energy level structure.

## Discussion

We have presented a study of the excited-state spin level structure of ${{{{{{\rm{V}}}}}}}_{{{{{{{{\rm{B}}}}}}}}}^{-}$ defects in hBN by performing spin-resonance optical spectroscopy. Our measurements establish the ES room-temperature zero-field longitudinal splitting *D*_es_ = 2.1 GHz, transverse splitting *E*_es_ = 74 MHz, and power-broadened linewidth of 154 MHz. We do not resolve a hyperfine splitting, which we expect is due to the power broadening that occurs at the large Rabi fields necessary to observe ES ESR. Using pulsed ODMR, we verify that the extra ESR features correspond to spin rotations in the ES, in agreement with other recent reports^43–45^. In addition, we observe agreement between magnetic-field-dependent PL measurements and a simple model of LACs in the GS and ES. Our temperature-dependent ESR measurements show that the ES ESR contrast around the *D*_es_ frequency not only persists at cryogenic temperatures, but also increases drastically. This and the accompanying linewidth reduction is well-explained by an increase in the ES lifetime.

Our temperature-dependent ODMR measurements indicate a lack of dynamic Jahn–Teller distortion effects, the well-known cause of fast orbital telegraphing in ^3^*E″* excited state systems like the diamond NV^−^ center that is quenched at low temperature. This can possibly be explained by large orbital energy splitting accompanying the symmetry-lowering which renders dynamic orbital averaging ineffective. The persistence of the ES ESR across the measured temperature range and enhanced transverse splitting are consistent with symmetry lowering from the intrinsic *D*_3*h*_ point-group to *C*_2*v*_. In addition, a study of the hyperfine splitting near the ELAC could shed light on the coupling to nuclear spins^31,46^, which could have applications in long-lived spin-based memories^47^ or quantum simulation^48^. Rapid progress is being made in integrating hBN defects into nano-photonic devices with waveguides^13^ and optical cavities^14,49^ to achieve high signal-to-noise ratios for sensing applications. We also envision using V${}_{{{{{{{{\rm{B}}}}}}}}}^{-}$ defects as quantum sensors^16^ for magnetization of layered out-of-plane magnets like CrI_3_ and CrBr_3_.

## Methods

### hBN device fabrication

Monocrystalline hBN was tap exfoliated into thin flakes (10–100 nm) and transferred onto silicon substrates with 285-nm-thick thermal oxide layers on top. The purpose of the thermal oxide layer is to increase the optical contrast for observing thin hBN flakes. The hBN flakes were irradiated with 2.5 keV He^+^ ions in a home-built ion implanter to create ${{{{{{\rm{V}}}}}}}_{{{{{{{{\rm{B}}}}}}}}}^{-}$ defects. With a flux of 1.6 × 10^11^ cm^−2^ per second for 10 min, the integrated dose density reaches ~10^14^ cm^−2^. To fabricate the microwave CPW device substrates, first a 3 μm Au film was deposited onto a sapphire substrate using a CVC SC4500 e-beam evaporation tool. Photoresist was spun and a GCA 6300 DSW 5x g-line wafer stepper tool was used to pattern the regions between the ground and central conductor planes. The exposed Au was then removed with a I_2_/KI Au etchant solution. The hBN flakes with spin active defects were transferred onto the CPW substrates using a stamp consisting of a thin polycarbonate (PC) film mounted on a supporting block of polydimethylsiloxane (PDMS) on a glass microscope slide. The glass slide is placed on a micropositioner in a home-built transfer station and the SiO_2_ substrate with hBN flakes is held in place on a heated stage with double-sided kapton tape, directly below the polymer stamp. The stamp is lowered until it makes contact with the substrate, upon which the temperature is increased to 80 ^∘^C, allowing flakes to adhere to the PC as it is lifted off the substrate. Then, the Au CPW substrate is placed on the heated stage and the flake is positioned above the central conductor of the waveguide and lowered until it makes contact with the Au. The heater is then set to 150 ^∘^C allowing the PC to melt off of the PDMS and adhere to the substrate. The glass slide with PDMS is then lifted and removed, and the sample is gently placed in a chloroform solution to dissolve the PC, leaving behind only the transferred hBN flake. Finally, we fix the device to our sample-holder using a thin layer of wax and use a West Bond 747630E tool to wire-bond the device to the sample-holder, which is then mounted into our cryostat for measurements.

### Experimental setup for ODMR

All measurements were performed with a home-built laser-scanning confocal microscope (Fig. 1b). A 532 nm laser (Dragon Lasers) is passed through an acousto-optic modulator (AOM) and focused onto the substrate through a window in a Janis He-flow cryostat using a 50x 0.7 NA Olympus microscope objective. The filtered PL emission is separated from the excitation laser with a beam-splitter followed by a 532 nm notch filter and 637 nm long-pass filter. The PL emission is then focused into a multimode fiber and coupled to an Excelitas avalanche photodiode (APD). The microwaves are generated by a Stanford Research Systems SG384 signal generator and amplified with either a Mini-Circuits ZVE-3W-83+ high-power amplifier or Mini-Circuits ZVA-183-S+ wideband amplifier before being transmitted into the cryostat. For CW ODMR measurements, a Stanford Research Systems DG645 digital delay generator (DDG) sends pulses to modulate the SG384 output as well as a Mini-Circuits ZYSWA-2-50DR switch that directs the APD counts to two channels on a National Instruments DAQ for normalization of the PL during ODMR frequency sweeps. For pulsed ODMR measurements, the DDG sends the pulse scheme described in the text and illustrated in Fig. 3c to the AOM, SG384, and RF switch.

### Reporting summary

Further information on research design is available in the Nature Research Reporting Summary linked to this article.

## Supplementary information

Supplementary Information

Reporting Summary

## Data Availability

All data presented in the paper, both processed and unprocessed, are available upon request.

## Code availability

Data fitting and plotting code was written in Python, and is available upon request.
