# Supplementary material for: Excited-state spin-resonance spectroscopy of VB− defect centers in hexagonal boron nitride
Source: Nat Commun. 2022 Jun 9;13:3233. doi: 10.1038/s41467-022-30772-z (PMC9184587; doi:10.1038/s41467-022-30772-z)
Supplement: Supplementary file 1 — Supplementary Information [file 41467_2022_30772_MOESM1_ESM.pdf]

Supplementary Information: Excited-state spin-resonance spectroscopy of V_B^- defect centers in hexagonal boron nitride

Nikhil Mathur[1,*], Arunabh Mukherjee[2,*], Xingyu Gao[3], Jialun Luo[4], Brendan A. McCullian[1], Tongcang Li[3,5], A. Nick Vamivakas[2,6,7,8,†] & Gregory D. Fuchs[1,9,†]

[1] *School of Applied and Engineering Physics, Cornell University, Ithaca, NY, USA*

[2] *The Institute of Optics, University of Rochester, Rochester, NY, USA*

[3] *Department of Physics and Astronomy, Purdue University, West Lafayette, IN, USA*

[4] *Department of Physics, Cornell University, Ithaca, NY, USA*

[5] *Elmore Family School of Electrical and Computer Engineering, Purdue University, West Lafayette, IN 47907, USA*

[6] *Materials Science, University of Rochester, Rochester, NY, USA*

[7] *Department of Physics and Astronomy, University of Rochester, Rochester, NY, USA*

[8] *Center for Coherence and Quantum Optics, University of Rochester, Rochester, NY, USA*

[9] *Kavli Institute at Cornell for Nanoscale Science, Ithaca, NY, USA*

**Denotes equal contribution*

†*E-mail: gdf9@cornell.edu, nick.vamivakas@rochester.edu*

Supplementary Note 1: Photoluminescence spectra of V_B^- ensembles

As shown in figure 1(b) in the main text, we excite the V_B^- defect ensemble with a 532 nm laser in a confocal microscope setup. The emitted photoluminescence (PL) spectrum is shown in Supplementary Figure 1. We note that there is no discernible sharp zero-phonon line (ZPL), but we do observe a broad fluorescence band centered around 800 nm which likely represents phonon-mediated emission. For ODMR measurements, we isolate the emission from the spin-active defects by filtering the PL with a 670 nm longpass filter.

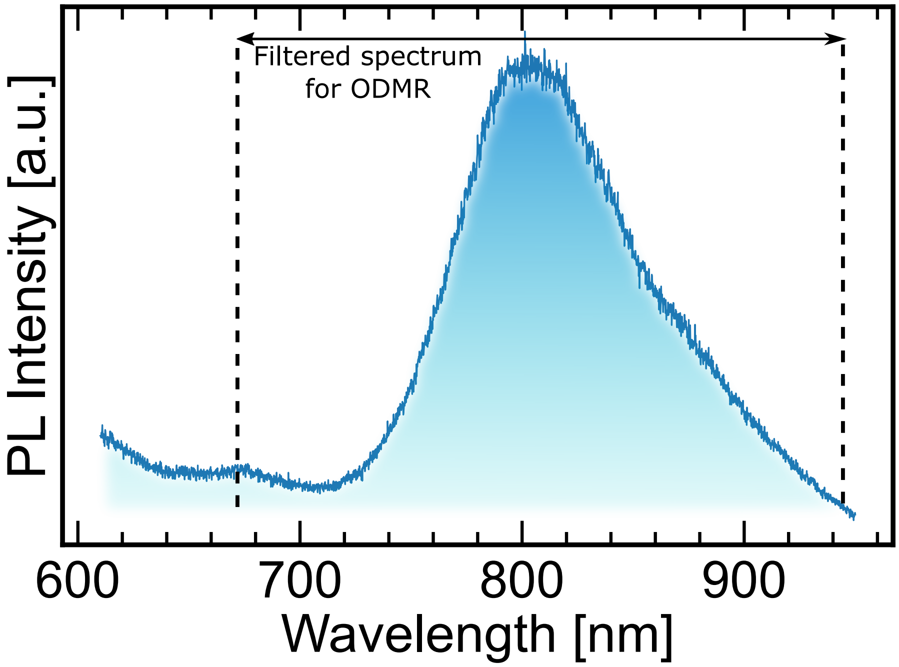

Supplementary Figure 1. Room-temperature optical spectrum of V_B^- defect emission

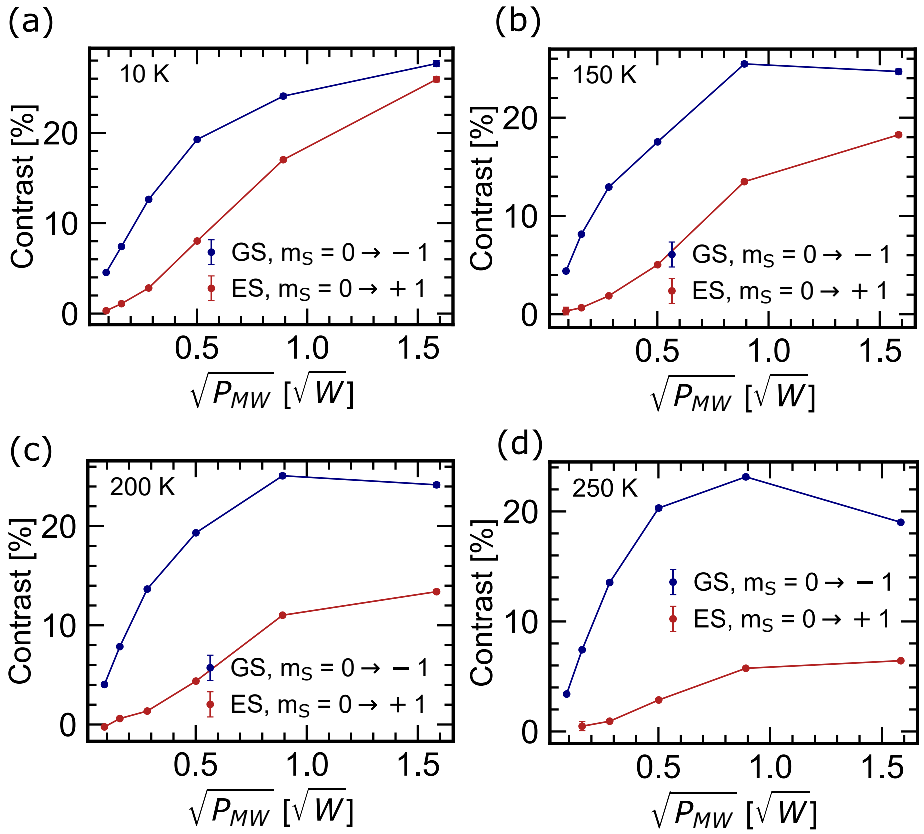

Supplementary Figure 2. Microwave power-dependent ODMR at temperatures **(a)** 10 K, **(b)** 150 K, **(c)** 200 K, and **(d)** 250 K.

Supplementary Note 2: Microwave power-dependent ODMR

Microwave power (P_{MW}) sweeps were performed at several temperatures from 10 K to 300 K, with an applied magnetic field of $B_0 = 450$ G. In Supplementary Figure 2, we show the the power-dependence of the ODMR contrast for both the GS (blue) and ES (red) spin transitions. The ES lifetime increases as the temperature is lowered[1], allowing the ES contrast at a given Rabi frequency to increase. As a result, the power saturation curve of the ES approaches the curve of the GS as the temperature is lowered. At 10 K, the ES/GS contrast ratio is close to 1 at the highest

microwave power in the sweep ($P_{\mathrm{MW}} \approx 2.51$ W) [also see Fig. 4(d) in the main text].

Supplementary Note 3: Temperature-dependent ES lifetime model

The observed temperature dependence of the ES ODMR contrast can be explained by considering the temperature dependence of the ES lifetime. As an example, we consider T = 10 K and 200 K where reported values are $\sim$1.95 ns and $\sim$1.6 ns respectively[1]. Because the lifetimes are short, it is reasonable to assume that the Rabi-rotation toward the $|\pm 1\rangle$ states is small. In this regime, we model the on-resonance temperature-dependent ODMR contrast, C as:

$$C(T) = \Delta C \sin^2\left(\frac{\Omega \tau_{es}(T)}{2}\right) \approx \Delta C \frac{\Omega^2 \tau_{es}(T)^2}{4} \tag{1}$$

where ΔC is the value of the ODMR contrast with full population transfer to the $|\pm 1\rangle$ states, Ω is the Rabi-frequency, and $\tau_{es}(T)$ is the temperature-dependent lifetime. From Supplementary Figure 2(a), we note the value of $C(10\ K) \sim 0.25$. Assuming that Ω is independent of temperature, we estimate that $C(200\ K) = C(10\ K)\left(\frac{\tau_{es}(200\ K)}{\tau_{es}(10\ K)}\right)^2 \approx 0.25\frac{1.6^2}{1.95^2} = 0.16$. Our observed value [Supplementary Figure 2(c)] is 0.14 which agrees closely with the calculations.

Supplementary Note 4: Level anti-crossing in the orbital excited and ground states

The essence of level anti-crossing (LAC) and spin-mixing in the orbital excited and ground states can be captured by studying the Hamiltonian:

$$\hat{H} = \mu_B g \vec{B}_0 \cdot \vec{\hat{S}} + hD\hat{S}_z^2 = \mu_B g B_{0z} \hat{S}_z cos\theta + \mu_B g B_{0x} \hat{S}_x sin\theta + hD\hat{S}_z^2 \tag{2}$$

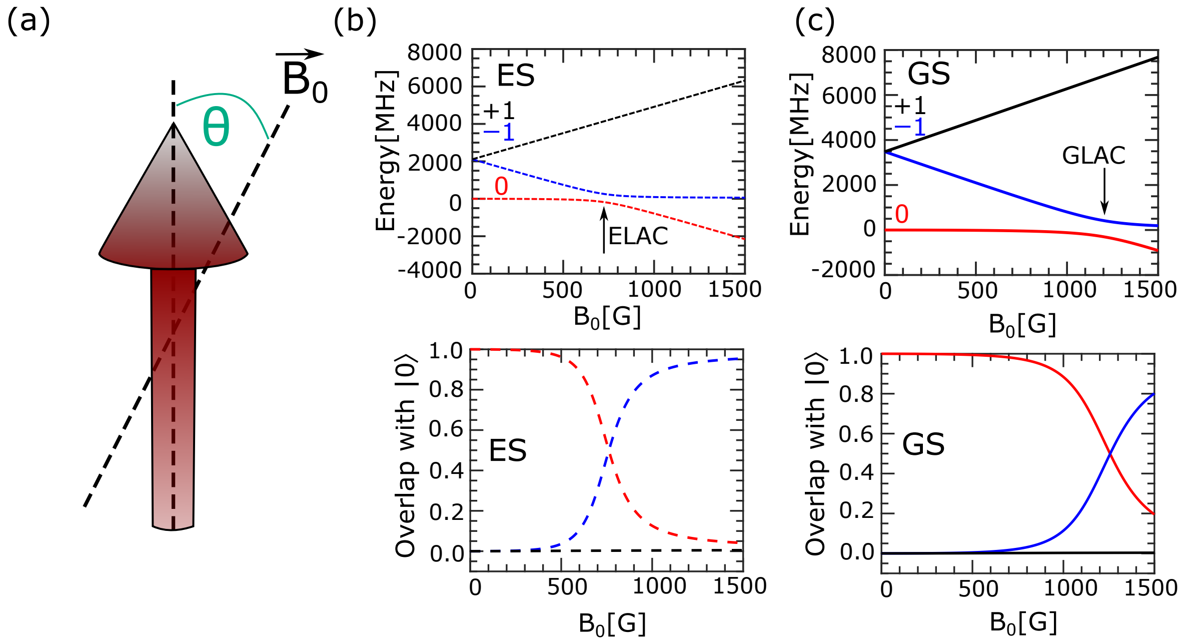

Supplementary Figure 3. Model of level anti-crossings (LACs) and spin-mixing

a) Magnetic field $\vec{B_0}$ applied at an angle θ with respect to the defect axis shown along the vertical direction. **b-c)** Level anti-crossing (top) and spin mixing (bottom) at $\theta = 6°$ for the orbital excited state (b) and ground state (c).

Here we have assumed that the g-factor of the defect is isotropic and have neglected the transverse splitting parameter $E \ll D$ at room temperature. Without loss of generality, we have also assumed that the applied field $\vec{B_0}$ lies in the x-z plane at an angle θ [Supplementary Figure 3(a)] with the defect axis along z. We show the spin eigen-energies and overlap of the eigenvectors with the $|0\rangle$ Zeeman sub-level in the orbital excited and ground states in Supplementary Figure 3(b) and (c) respectively. The energy eigenvalue plots show that the LACs occur at $\sim$750 G and $\sim$1240G in the excited and ground states, which match very well with our observations. At the LAC, the eigenvectors overlap with the $|0\rangle$ and $|-1\rangle$ equally, confirming spin-mixing.

In the main text, we have shown that the magnitude of the photo-luminescence (PL) intensity drops at the LAC fields. We attribute this effect to mixing of $|0\rangle$ with $|-1\rangle$ resulting in an increase in non-radiative relaxation. The magnetic field-dependent PL is governed by the steady-state populations of the $|0\rangle$ and $|-1\rangle$ levels which are dependent on the competing phenomena of spin-polarization from optical pumping and spin-mixing[2]. A Lindbladian approach with appropriate optical pumping, inter-system crossing, and spin-relaxation rates is needed to fully explain our observations which is beyond the scope of this work.

Supplementary References

1. Liu, W. *et al.* Temperature-dependent energy-level shifts of spin defects in hexagonal boron nitride. *ACS Photonics* **8**, 1889–1895 (2021). URL `https://doi.org/10.1021/acsphotonics.1c00320`. `https://doi.org/10.1021/acsphotonics.1c00320`.

2. Epstein, R. J., Mendoza, F. M., Kato, Y. K. & Awschalom, D. D. Anisotropic interactions of a single spin and dark-spin spectroscopy in diamond. *Nature Physics* **1**, 94–98 (2005). URL `https://doi.org/10.1038/nphys141`.
